# Late preterm birth and growth trajectories during childhood: a linked retrospective cohort study

**DOI:** 10.1186/s12887-023-04257-x

**Published:** 2023-09-08

**Authors:** Yulika Yoshida-Montezuma, David Kirkwood, Branavan Sivapathasundaram, Charles D. G. Keown-Stoneman, Russell J. de Souza, Teresa To, Cornelia M. Borkhoff, Catherine S. Birken, Jonathon L. Maguire, Hilary K. Brown, Laura N. Anderson, on behalf of the TARGet Kids! Collaboration, Christopher Allen, Danielle D’Annunzio, Mateenah Jaleel, Natricha Levy McFarlane, Jessica A. Omand, Sharon Thadani, Mary Aglipay, Imaan Bayoumi, Cornelia M. Borkhoff, Sarah Carsley, Alice Charach, Katherine Cost, Curtis D’Hollander, Anne Fuller, Laura Kinlin, Michaela Kucab, Patricia Li, Pat Parkin, Nav Persaud, Sarah Rae, Izabela Socynska, Shelley Vanderhout, Leigh Vanderloo, Peter Wong, Piyumi Konara Mudiyanselage, Xuedi Li, Jenny Liu, Michelle Mitchell, Nusrat Zaffar, Tiffany Bondoc, Trudy-Ann Buckley, Pamela Ruth Flores, Kardelen Kurt, Sangeetha Loganathan, Tarandeep Mali, Laurie Thompson, Jennifer Batten, Jennifer Chan, John Clark, Amy Craig, Kim De Castris-Garcia, Sharon Dharman, Sarah Kelleher, Salimah Nasser, Tammara Pabon, Michelle Rhodes, Rafael Salsa, Julie Skelding, Daniel Stern, Kerry Stewart, Erika Sendra Tavares, Shannon Weir, Maria Zaccaria-Cho, Magdalena Janus, Eric Duku, Caroline Reid-Westoby, Patricia Raso, Amanda Offord, Emy Abraham, Sara Ali, Kelly Anderson, Gordon Arbess, Jillian Baker, Tony Barozzino, Sylvie Bergeron, Gary Bloch, Joey Bonifacio, Ashna Bowry, Caroline Calpin, Douglas Campbell, Sohail Cheema, Brian Chisamore, Evelyn Constantin, Karoon Danayan, Paul Das, Viveka De Guerra, Mary Beth Derocher, Anh Do, Kathleen Doukas, Anne Egger, Allison Farber, Amy Freedman, Sloane Freeman, Sharon Gazeley, Karen Grewal, Charlie Guiang, Curtis Handford, Laura Hanson, Leah Harrington, Sheila Jacobson, Lukasz Jagiello, Gwen Jansz, Paul Kadar, Lukas Keiswetter, Tara Kiran, Holly Knowles, Bruce Kwok, Piya Lahiry, Sheila Lakhoo, Margarita Lam-Antoniades, Eddy Lau, Denis Leduc, Fok-Han Leung, Alan Li, Patricia Li, Roy Male, Aleks Meret, Elise Mok, Rosemary Moodie, Katherine Nash, James Owen, Michael Peer, Marty Perlmutar, Navindra Persaud, Andrew Pinto, Michelle Porepa, Vikky Qi, Noor Ramji, Danyaal Raza, Katherine Rouleau, Caroline Ruderman, Janet Saunderson, Vanna Schiralli, Michael Sgro, Shuja Hafiz, Farah Siam, Susan Shepherd, Cinntha Srikanthan, Carolyn Taylor, Stephen Treherne, Suzanne Turner, Fatima Uddin, Meta van den Heuvel, Thea Weisdorf, Peter Wong, John Yaremko, Ethel Ying, Elizabeth Young, Michael Zajdman, Esmot Ara Begum, Peter Juni, Gurpreet Lakhanpal, Gerald Lebovic, Ifeayinchukwu (Shawn) Nnorom, Marc Denzel Nunez, Audra Stitt, Kevin Thorpe, Raya Assan, Homa Bondar, George S. Charames, Andrea Djolovic, Chelsea Gorscak-Dunn, Mary Hassan, Rita Kandel, Michelle Rodrigues

**Affiliations:** 1 Department of Health Research Methods, Evidence, and Impact, McMaster University, 1280 Main Street W, Hamilton, ON L8S 4L8 Canada; 2 ICES, McMaster University, Hamilton, ON Canada; 3 Dalla Lana School of Public Health, University of Toronto, Toronto, ON Canada; 4 Li Ka Shing Knowledge Institute, Unity Health Toronto, Toronto, ON Canada; 5 Department of Nutritional Sciences, Faculty of Medicine, University of Toronto, Toronto, ON Canada; 6 Population Health Research Institute, Hamilton Health Sciences Corporation, Hamilton, ON Canada; 7 ICES, Sunnybrook Health Sciences Centre, Toronto, ON Canada; 8 Child Health Evaluative Sciences, The Hospital for Sick Children Research Institute, Toronto, ON Canada; 9 Women’s College Research Institute, Toronto, ON Canada; 10 Division of Pediatric Medicine, The Hospital for Sick Children, Toronto, ON Canada; 11 Department of Pediatrics, St. Michael’s Hospital, Unity Health Toronto, Toronto, ON Canada; 12 Department of Pediatrics, Faculty of Medicine, University of Toronto, Toronto, ON Canada; 13 Department of Health & Society, University of Toronto Scarborough, Toronto, ON Canada

**Keywords:** Late preterm, Gestational age, Growth trajectory, Height, Weight, Children

## Abstract

**Background:**

Evidence suggests that accelerated postnatal growth in children is detrimental for adult cardiovascular health. It is unclear whether children born late preterm (34–36 weeks) compared to full term (≥ 39 weeks), have different growth trajectories. Our objective was to evaluate the association between gestational age groups and growth trajectories of children born between 2006–2014 and followed to 2021 in Ontario, Canada.

**Methods:**

We conducted a retrospective cohort study of children from singleton births in TARGet Kids! primary care network with repeated measures of weight and height/length from birth to 14 years, who were linked to health administrative databases. Piecewise linear mixed models were used to model weight (kg/month) and height (cm/month) trajectories with knots at 3, 12, and 84 months. Analyses were conducted based on chronological age.

**Results:**

There were 4423 children included with a mean of 11 weight and height measures per child. The mean age at the last visit was 5.9 years (Standard Deviation: 3.1). Generally, the more preterm, the lower the mean value of weight and height until early adolescence. Differences in mean weight and height for very/moderate preterm and late preterm compared to full term were evident until 12 months of age. Weight trajectories were similar between children born late preterm and full term with small differences from 84–168 months (mean difference (MD) -0.04 kg/month, 95% CI -0.06, -0.03). Children born late preterm had faster height gain from 0–3 months (MD 0.70 cm/month, 95% CI 0.42, 0.97) and 3–12 months (MD 0.17 cm/month, 95% CI 0.11, 0.22).

**Conclusions:**

Compared to full term, children born late preterm had lower average weight and height from birth to 14 years, had a slightly slower rate of weight gain after 84 months and a faster rate of height gain from 0–12 months. Follow-up is needed to determine if growth differences are associated with long-term disease risk.

**Supplementary Information:**

The online version contains supplementary material available at 10.1186/s12887-023-04257-x.

## Introduction

Late preterm birth, defined as birth between 34 and 36 weeks gestation, accounts for 75% of all preterm births and this percentage has been rising since 1990 [1]. Increases in obstetrical interventions, such as induced delivery and caesarean section, are important contributing factors to the increase in late preterm births but much of the reason remains unexplained [2–4]. Late preterm newborns are not as physiologically or metabolically mature as full term newborns and late preterm birth may lead to adverse health outcomes across the life course [5]. A recent systematic review of the association between late preterm birth and cardiometabolic health outcomes found that children and adults born late preterm compared to full term were at increased risk of diabetes (pooled adjusted relative risk 1.24 (95% confidence interval (CI) 1.17, 1.32) from 9 studies), and hypertension (pooled adjusted relative risk 1.21 (95% CI 1.13, 1.30) from 11 studies), but had lower BMI z-scores (standardized mean difference -0.38 (95% CI -0.67, -0.09) from 5 studies) [6]. Further, a retrospective cohort study assessing the association between preterm gestational age and childhood cardiometabolic risk (CMR) score found that those born late preterm had higher CMR compared with those born full term [7]. As the CMR score tracks risk from childhood into adulthood [8–10], these findings suggest that late preterm birth may be an important risk factor for cardiometabolic disorders later in life. Although the risk of late preterm birth on later adverse outcomes is small on the individual level, a large number of children born late preterm are reaching young adulthood when the incidence of cardiometabolic diseases may increase [11].

Growth during infancy and childhood are important indicators of child health and development and have an important influence on health later in life [12]. Previous studies have reported that accelerated postnatal growth in weight could be detrimental for adult cardiovascular health [13–15]. However, most growth trajectory research is limited to children born full term [16, 17] or overall preterm (defined as < 37 weeks gestation) [18, 19] with few studies investigating growth trajectories by gestational age groups, including late preterm birth. Understanding the effects of late preterm birth and growth trajectories can elucidate the link between late preterm birth and cardiometabolic outcomes for this potentially high-risk population.

The primary objective of this study was to evaluate the association between gestational age groups (very/moderate preterm (< 34 weeks), late preterm (34–36 weeks), early term (37–38 weeks) compared to full term (≥ 39 weeks) and trajectories of weight and height from early infancy to mid-childhood. Our secondary objective was to determine whether weight and height growth rates differed during each growth period for gestational age categories by sex.

## Methods

### Study population and design

We conducted a retrospective cohort study of children born between April 1, 2006, and March 31, 2014, followed until 2021, participating in The Applied Research Group for Kids (TARGet Kids!) primary care practice-based research network in Toronto, Canada [20]. Inclusion criteria for the TARGet Kids! cohort include children < 6 years of age and receiving primary healthcare at a TARGet Kids! participating site. Exclusion criteria include health conditions affecting growth (e.g., failure to thrive, cystic fibrosis), any acute or chronic conditions (other than asthma and high functioning autism), severe developmental delay and families unable to communicate in English [20]. Data from children in TARGet Kids! who had weight and height (or length if < 2 years) measured from birth to 14 years were deterministically linked using individual Ontario Health Insurance Plan (OHIP) numbers (98% success) to population-based health administrative data at ICES (Ontario, Canada). ICES is an independent, non-profit research institute whose legal status under Ontario’s health information privacy law allows it to collect and analyze health care and demographic data, without requiring individual participant consent, for the purpose of health system evaluation and improvement. We used the MOMBABY dataset at ICES, which is derived from hospital discharge abstracts, to identify maternal-newborn records for all hospital births (98% of births in Ontario) [21]. The MOMBABY dataset was then linked with the Better Outcomes Registry and Network (BORN), a province-wide registry of all births in Ontario, Canada, for more detailed clinical perinatal data [22]. For this study, perinatal data from BORN were available at ICES for children born between April 1, 2006, and March 31, 2014. Ethics approval was granted by the Hospital for Sick Children, Unity Health Toronto, and the Hamilton Integrated Research Ethics Boards. All methods were carried out in accordance with relevant guidelines and regulations. Informed consent was obtained from all subjects and/or their legal guardian(s).

### Exposures

The primary exposure was gestational age at birth which was calculated based on the best clinical estimate of gestation using a combination of ultrasound and last menstrual period-based estimates [23]. For children born in hospitals, gestational age was identified from the MOMBABY dataset while BORN data were used to identify gestational age for children born through home births. Gestational age was analyzed categorically with four groups defined a priori: very/moderate preterm (< 34 weeks), late preterm (34–36 weeks), early term (37–38 weeks), and full term (≥ 39 weeks, used as the reference group) [24, 25].

In this study, the very/moderate preterm group was defined as < 34 weeks because it included mostly children born between 32–34 weeks with a small number of children born < 32 weeks. Children born less than 32 weeks of age (considered very preterm) are generally excluded from TARGet Kids! at enrollment. However, some parents or caregivers did not report their child’s gestational age on the initial self-reported questionnaire, and these children were included. Once linked to administrative data, we found that 58 children < 32 weeks were included in the cohort, and we kept them in the analysis.

### Outcomes

The primary outcomes were the rate of weight change (kg/month) and rate of height change (cm/month) between 0–14 years identified from TARGet Kids! data. Children with only one measure of weight or height were excluded. Histograms were created for weight and height and extreme outliers that represented implausible values were removed (i.e., 8 kg (after 30 months) > weight > 75 kg or 30 cm > height > 200 cm). For the weight model, birthweight was included as the initial measurement. Weight and height were measured by trained TARGet Kids! research staff members using standardized instruments [26] and were analyzed continuously. Our primary interest was to characterize and understand the differences in growth in children born late preterm compared to full term over a range of ages; therefore, our primary outcome was the unstandardized growth measures which we describe by chronological age and sex. The World Health Organization growth reference standards are recommended for full-term children, and while they are often used by correcting preterm infants to term age, this is usually only done until up to 2 or 3 years of age but not beyond and in our study children were followed until up to 14 years of age [27]. As a secondary analysis in this paper, we included the WHO standardized values but given the range of age for the outcomes and the primary goal of this research study to understand the impact of preterm on growth measures, we did not correct for gestational age. Z-standardized measures of weight, height, and BMI were adjusted for child’s age and sex using the World Health Organization recommendations. Biologically implausible values were identified using cut-points proposed by the World Health Organization (values with z-scores < -5 or > 5) and removed. The secondary outcomes of interest were the rate of change in zweight, zheight, and zBMI per month.

### Confounding variables

Confounding variables were selected a priori guided by previous literature and included maternal, child, and sociodemographic characteristics hypothesized to be associated with gestational age at birth and child growth, but not on the causal path between them [6]. The included confounders were maternal age at delivery, maternal ethnicity, family income, child age at outcome, and child sex. A table describing the data source and operationalization of variables is provided in Supplemental Table 1.

### Statistical analysis

Repeated measures of weight and height were used to estimate the rate of weight and height change for each gestational age group through piecewise linear mixed models with random intercepts. Knots were determined by using a locally estimated scatterplot smoothing (LOESS) curve to visually assess the approximate locations (age in months) at which growth rates substantially changed in slope in our cohort. Based on the LOESS curve, we fit knot points at 3, 12, and 84 months of age which also aligned with knot points in other growth studies [12]. Sensitivity analyses were conducted to determine: 1) whether changing the value of knots (different months) or 2) increasing the number of knots based on literature and data distribution changed the results [12]. The resulting model with the best fit (4 knots) was selected based on model fit statistics (e.g., Akaike information criterion (AIC) and the Bayesian information criterion (BIC)) and the estimated results were compared to results from knots determined through the LOESS curve (3 knots). As the model fit between the 3 knot and 4 knot model were similar in terms of fit statistics, estimates, and standard errors, we chose the 3-knot model for parsimony (Supplemental Tables 2 and 3). We estimated growth rates for each growth period (period between knot points) comparing the gestational age categories to full term by taking the difference between the beta coefficients. Contrasts were used to estimate the difference in slopes between gestational age categories and obtain corresponding confidence intervals. As a secondary analysis, sex-stratified rates of growth were examined to determine whether growth rates during each growth period differed for boys and girls. All analyses were adjusted for maternal age at delivery, maternal ethnicity, child age, child sex, and family income.

Missing data for each covariate were imputed using multiple imputation and a total of 10 imputed data sets with the PROC MI procedure and pooled using Rubin’s rules with PROC MIANALYZE. All analyses were conducted using SAS, version 9.4.

## Results

There were 10,897 children overall in TARGet Kids! but 4990 of them had a birthdate outside of the eligible range of this study. Of the 5907 potentially eligible children, 108 were not included in the analysis because they were not eligible for Ontario’s health care plan, 770 were excluded because of insufficient weight/height measures, 311 were missing gestational age data, and 295 children were part of multiple births. The final study cohort consisted of 4423 (75%) of the eligible children (Supplemental Fig. 1). The mean number of growth measures per child was 11. Of 4423 children, 2320 (52%) were male and the mean age at the last visit (i.e., the average follow-up time) was 5.9 years (Standard Deviation: 3.1). Late preterm children were more likely than full term children to be male and have lower family income; and their mothers were more likely to be older and be of non-European ethnicity. The characteristics of the study population, overall and by gestational age categories, are presented in Table 1.

**Table 1:** Characteristics of study participants (*N* = 4423) by gestational age at birth for the outcome of weight/height

Characteristic	N (%)				
	**Overall *N* = 4423**	**Very/moderate preterm (< 34 weeks)** ***N* = 133 (3.0%)**	**Late preterm (34–36 weeks)** ***N* = 291 (6.6%)**	**Early term (37–38 weeks)** ***N* = 1156 (26.1%)**	**Full term (≥ 39 weeks)** ***N* = 2843 (64.3%)**
**Maternal**					
Mean (SD) age (years)	33.2 (4.6)	33.0 (4.6)	33.3 (4.8)	33.6 (4.7)	33.1 (4.6)
Missing	122 (3%)	4–8 (3–6%)	1–5 (< 2%)	34 (3%)	78 (3%)
Ethnicity					
African/Arab/Latin American/Mixed	687 (16%)	28 (21%)	50 (17%)	185 (16%)	424 (15%)
East/Southeast/South Asian	784 (18%)	23 (17%)	61 (21%)	237 (21%)	463 (16%)
European	2476 (56%)	61 (46%)	139 (48%)	612 (53%)	1664 (59%)
Missing	476 (11%)	21 (16%)	41 (14%)	122 (11%)	292 (10%)
**Child**					
Mean (SD) age (years) at last visit	5.9 (3.1)	5.7 (3.5)	5.5 (3.1)	5.9 (3.2)	5.9 (3.1)
Median (Q1, Q3) age (years) at last visit	6 (3, 8)	5 (2, 8)	5 (3, 8)	6 (3, 8)	6 (4, 8)
Sex					
Female	2103 (48%)	63 (47%)	133 (46%)	540 (47%)	1367 (48%)
Male	2320 (52%)	70 (53%)	158 (54%)	616 (53%)	1476 (52%)
**Sociodemographic**					
Family income					
< $50, 000	486 (11%)	31 (23%)	45 (16%)	136 (12%)	274 (10%)
$50, 000 to $99, 999	1091 (25%)	30 (23%)	66 (23%)	292 (25%)	703 (25%)
$100, 000 to $149, 999	364 (8%)	7 (5%)	33 (11%)	104 (9%)	220 (8%)
$150, 000 or more	1899 (43%)	51 (38%)	113 (39%)	462 (40%)	1273 (45%)
Missing	583 (13%)	14 (11%)	34 (12%)	162 (14%)	373 (13%)

### Weight trajectories

**Fig. 1:**
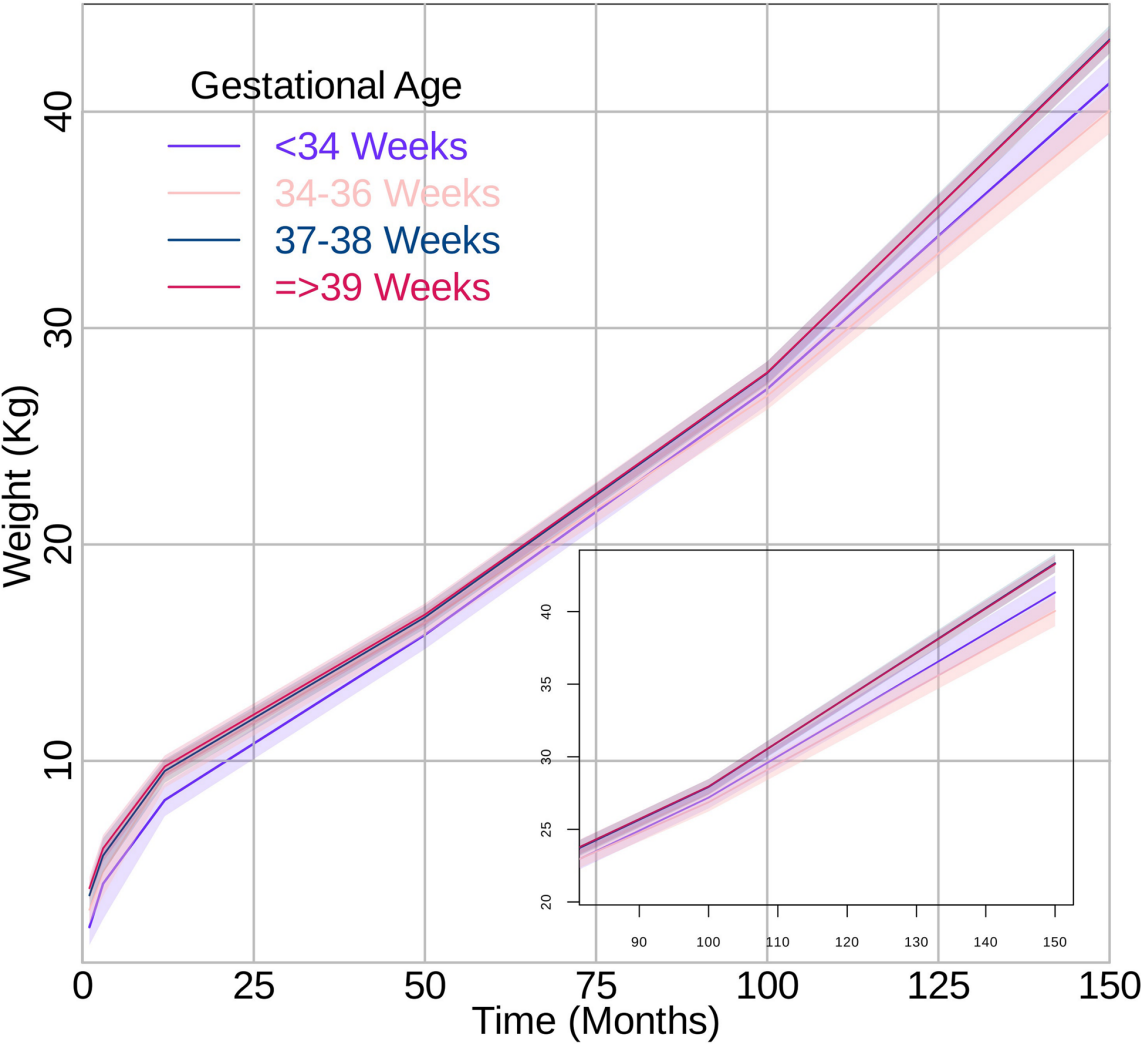
Growth curves for weight in kg per month from 0 to 150 months by gestational age categories. The lines represent the growth curve, and the shaded areas represent the 95% confidence intervals. The box represents a superimposed area of the growth curve between 75 to 150 months

Children born late preterm had lower weight compared to full term through the age range studied (0–14 years). These results are presented in Fig. 1 and Supplemental Table 4. For late preterm compared to full term, the rate of weight growth was similar from 3–12 months (MD 0.08 kg/month 95% CI -0.04, 0.20) and the rate of weight growth was slower from 84–168 months (MD -0.04 kg/month 95% CI -0.06, -0.03). However, there was little to no evidence of differences in the rate of weight growth from 0–3 months and 12–84 months. These results are presented in Table 2.

**Table 2:** Mean differences in weight growth rates (kg per month) and height growth rates (cm per month) by gestational age categories during each growth period for study participants (*N* = 4423)

Growth period	Growth rate (95% CI) very/moderate preterm	Mean difference (95% CI) between very/moderate preterm vs. full term	Growth rate (95% CI) late preterm	Mean difference (95% CI) between late preterm vs. full term	Growth rate (95% CI) early term	Mean difference (95% CI) between early term vs. full term	Growth rate (95% CI) full term
**Weight (kg/month)**							
0–3 months	1.00 (0.48, 1.53)	0.08 (-0.46, 0.62)	0.87 (0.54, 1.20)	-0.05 (-0.41, 0.30)	0.92 (0.72, 1.13)	0.00 (-0.24, 0.23)	0.92 (0.80, 1.04)
3–12 months	0.43 (0.25, 0.61)	0.01 (-0.18, 0.20)	0.50 (0.39, 0.62)	0.08 (-0.04, 0.20)	0.43 (0.36, 0.50)	0.01 (-0.07, 0.09)	0.42 (0.38, 0.46)
12–84 months	0.20 (0.19, 0.21)	0.02 (0.01, 0.03)	0.18 (0.18, 0.19)	0.00 (-0.01, 0.01)	0.19 (0.18, 0.19)	0.00 (0.00, 0.01)	0.18 (0.18, 0.19)
84–168 months	0.28 (0.26, 0.30)	-0.02 (-0.04, -0.01)	0.26 (0.25, 0.28)	-0.04 (-0.06, -0.03)	0.31 (0.30, 0.32)	0.00 (-0.01, 0.01)	0.31 (0.30, 0.31)
**Height (cm/month)**							
0–3 months	4.57 (3.87, 5.27)	0.99 (0.29, 1.69)	4.28 (4.02, 4.55)	0.70 (0.42, 0.97)	3.90 (3.78, 4.03)	0.32 (0.17, 0.46)	3.59 (3.50, 3.67)
3–12 months	2.25 (2.17, 2.34)	0.49 (0.41, 0.58)	1.93 (1.87, 1.98)	0.17 (0.11, 0.22)	1.81 (1.78, 1.84)	0.05 (0.02, 0.08)	1.76 (1.74, 1.78)
12–84 months	0.67 (0.66, 0.68)	0.04 (0.02, 0.05)	0.64 (0.63, 0.64)	0.00 (0.00, 0.01)	0.63 (0.63, 0.64)	0.00 (0.00, 0.00)	0.63 (0.63, 0.63)
84–168 months	0.44 (0.42, 0.46)	0.02 (0.00, 0.04)	0.43 (0.41, 0.45)	0.01 (-0.01, 0.03)	0.44 (0.43, 0.45)	0.02 (0.01, 0.03)	0.42 (0.41, 0.43)

Children born very/moderately preterm consistently had lower weight than full term (Supplemental Table 4). There was no evidence of differences in growth rates from 0–3 months and 3–12 months; however, growth rates were faster from 12–84 months (MD 0.02 kg/month 95% CI 0.01, 0.03) but slightly slower from 84–168 months (MD -0.02 kg/month 95% CI -0.04, -0.01) for very/moderate preterm compared to full term (Table 2).

Children born early term consistently had similar weight and growth rates for weight to full term (Supplemental Table 4; Table 2).

### Height trajectories

**Fig. 2:**
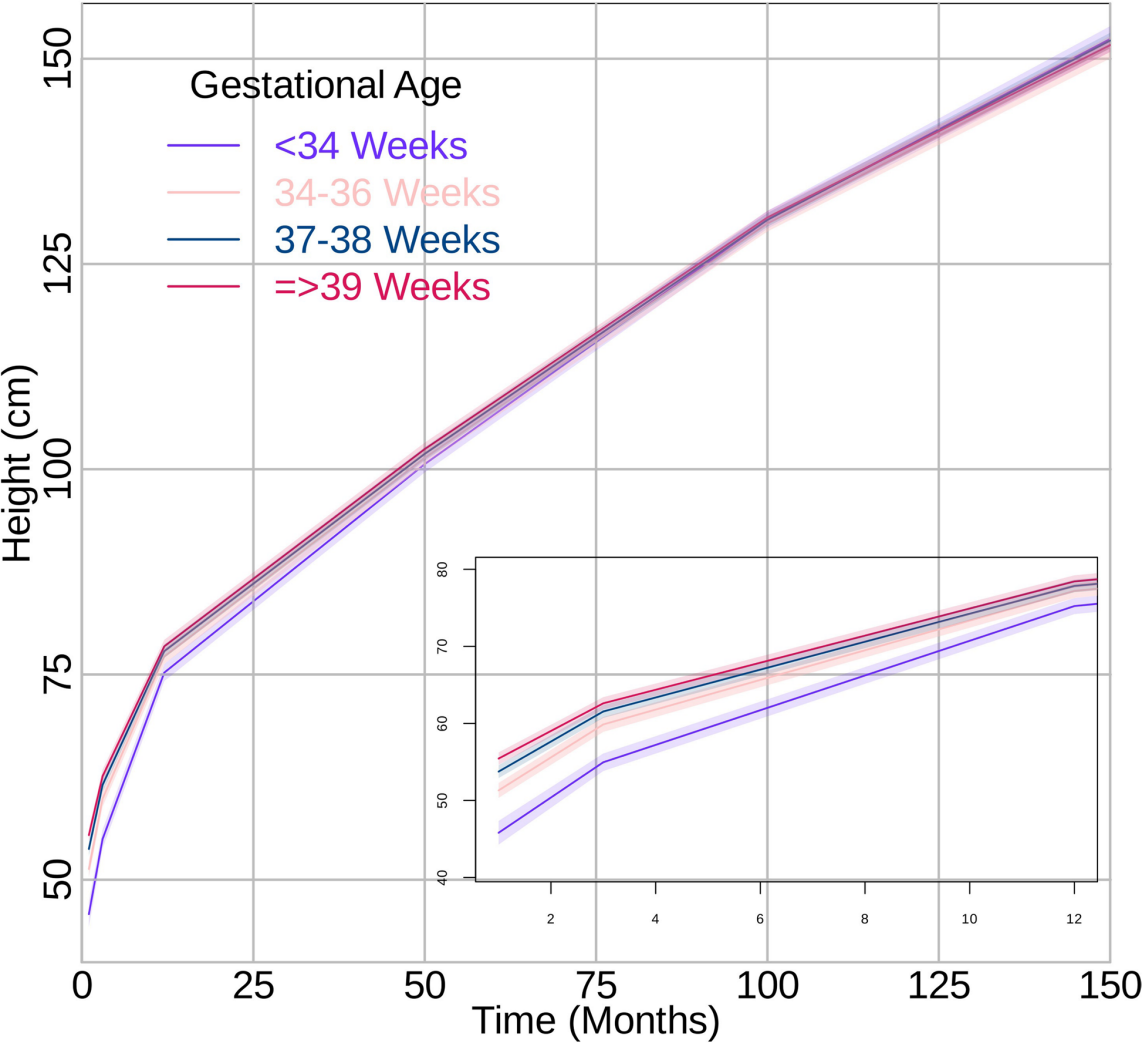
Growth curves for height in cm per month from 0 to 150 months by gestational age categories. The lines represent the growth curve, and the shaded areas represent the 95% confidence intervals. The box represents a superimposed area of the growth curve between 0 to 12 months

Children born late preterm consistently had lower height than full term. These results are presented in Fig. 2 and Supplemental Table 5. For late preterm compared to full term, the rate of height growth was faster from 0–3 months (MD 0.70 cm/month 95% CI 0.42, 0.97) and 3–12 months (MD 0.17 cm/month 95% CI 0.11, 0.22). There was no evidence of differences after 12 months. These results are presented in Table 2.

Children born very/moderate preterm had lower height than full term children until between 100-150 months of age when trajectories crossed, and very/moderate preterm children showed higher height (Fig. 2; Supplemental Table 5). Further, for very/moderate preterm compared to full term, the rate of height growth was faster at all ages (Table 2). The mean difference in the rate of height growth was 0.99 cm/month (95% CI 0.29, 1.69) from 0–3 months, 0.49 cm/month (95% CI 0.41, 0.58) from 3–12 months, 0.04 cm/month (95% CI 0.02, 0.05) from 12–84 months and 0.02 cm/month (95% CI 0.00, 0.04) from 84–168 months.

Children born early term consistently had similar heights to full term from 3 months of age onward (Supplemental Table 5). The growth rate for height was slightly faster from 0–3 months (MD 0.32 cm/month 95% CI 0.17, 0.46), 3–12 months (MD 0.05 cm/month 95% CI 0.02, 0.08), and 84–168 months (MD 0.02 cm/month 95% CI 0.01, 0.03) but there was no evidence of differences between 12–84 months for early term compared to full term (Table 2).

### Z-Standardized trajectories

For children born very/moderate preterm, late preterm, and early term compared to full term, differences in zweight (Supplemental Fig. 2), zheight (Supplemental Fig. 3), and zBMI (Supplemental Fig. 4) and rates of growth were evident. A table describing the z-standardized growth rates and mean differences compared to full term is provided in Supplemental Table 6.

### Secondary analysis

**Fig. 3:**
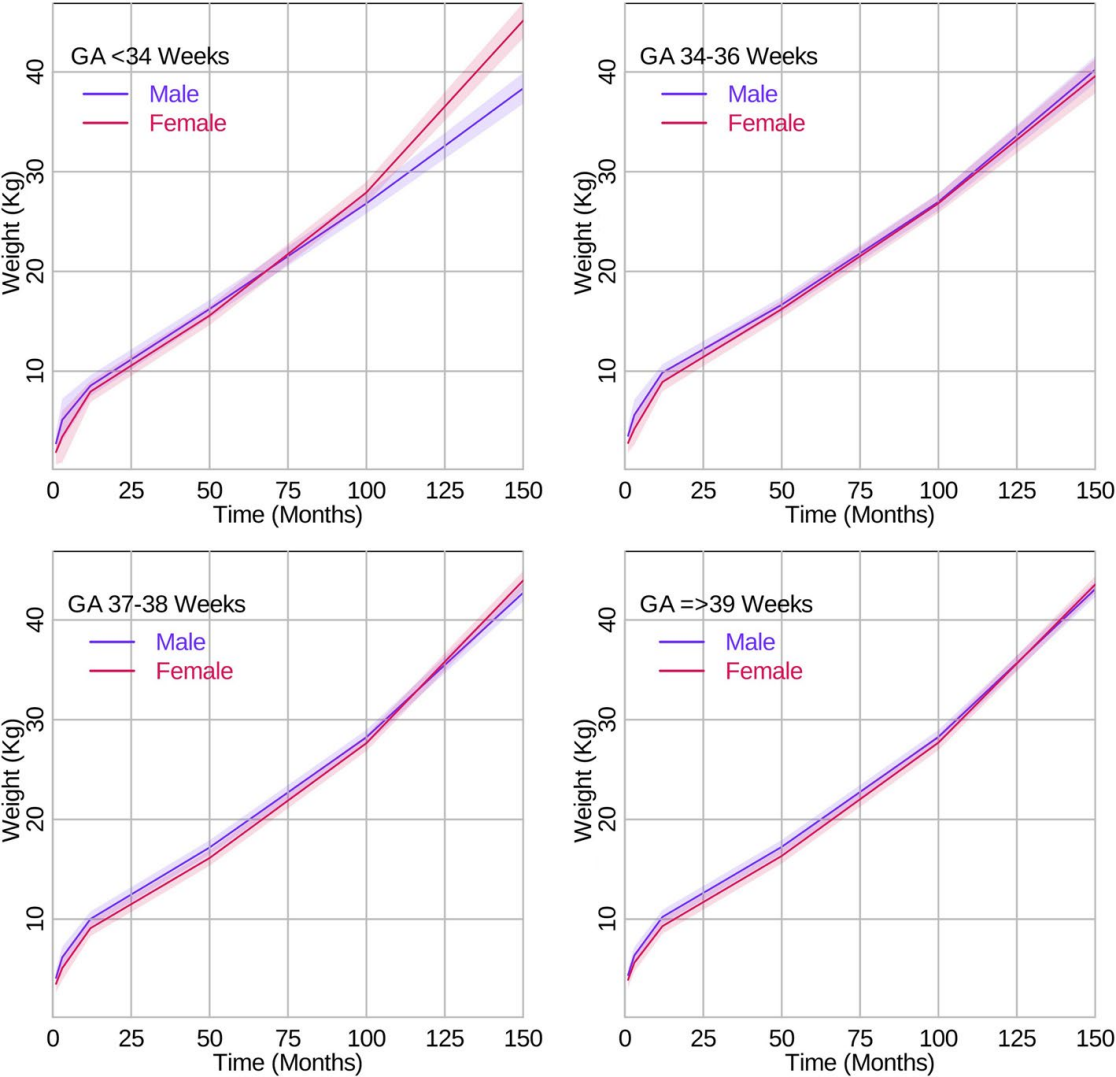
Growth curves for weight trajectories in kg per month from 0 to 150 months by sex and gestational age categories. The lines represent the growth curve, and the shaded areas represent the 95% confidence intervals

Regarding the rate of weight growth, for late preterm compared to full term, slight differences by sex were evident after 84 months where boys grew less slowly than girls. These results are presented in Fig. 3 and Table 3. Differences in the rate of weight growth between girls and boys were more evident when comparing very/moderate preterm to full term where boys (MD -0.07 kg/month, 95% CI -0.09, -0.04) had a slower rate of growth in weight from 84–168 months than girls (MD 0.03 kg/month, 95% CI 0.00, 0.05).

**Table 3:** Mean differences in weight growth rates (kg per month) and height growth rates (cm per month) by sex for gestational age categories during each growth period

Growth period	Mean difference (95% CI) between very/moderate preterm vs. full term		Mean difference (95% CI) between late preterm vs. full term		Mean difference (95% CI) between early term vs. full term	
Weight (kg/month)						
	Girls	Boys	Girls	Boys	Girls	Boys
0–3 months	-0.09 (-0.94, 0.77)	0.19 (-0.50, 0.89)	-0.15 (-0.66, 0.35)	0.05 (-0.44, 0.55)	-0.07 (-0.43, 0.30)	0.04 (-0.27, 0.35)
3–12 months	0.09 (-0.20, 0.38)	-0.05 (-0.29, 0.19)	0.12 (-0.06, 0.29)	0.04 (-0.13, 0.21)	0.04 (-0.09, 0.16)	0.00 (-0.11, 0.10)
12–84 months	0.02 (0.00, 0.03)	0.02 (0.00, 0.03)	0.01 (-0.00, 0.02)	-0.01 (-0.02, 0.00)	0.00 (-0.01, 0.01)	0.00 (0.00, 0.01)
84–168 months	0.03 (0.00, 0.05)	-0.07 (-0.09, -0.04)	-0.06 (-0.09, -0.04)	-0.03 (-0.05, -0.01)	0.01 (0.00, 0.02)	-0.01 (-0.02, 0.00)
Height (cm/month)						
0–3 months	0.80 (-0.17, 1.76)	1.18 (0.15, 2.21)	0.51 (0.09, 0.93)	0.78 (0.41, 1.15)	0.26 (0.05, 0.47)	0.34 (0.13, 0.54)
3–12 months	0.55 (0.43, 0.67)	0.45 (0.33, 0.57)	0.16 (0.08, 0.25)	0.17 (0.09, 0.25)	0.06 (0.01, 0.10)	0.05 (0.00, 0.09)
12–84 months	0.02 (0.01, 0.04)	0.05 (0.03, 0.06)	0.01 (0.00, 0.02)	0.00 (-0.01, 0.01)	0.00 (-0.01, 0.01)	0.00 (-0.01, 0.01)
84–168 months	0.05 (0.02, 0.09)	0.00 (-0.03, 0.03)	-0.06 (-0.09, -0.03)	0.05 (0.03, 0.08)	0.02 (0.01, 0.03)	0.01 (0.00, 0.03)

**Fig. 4:**
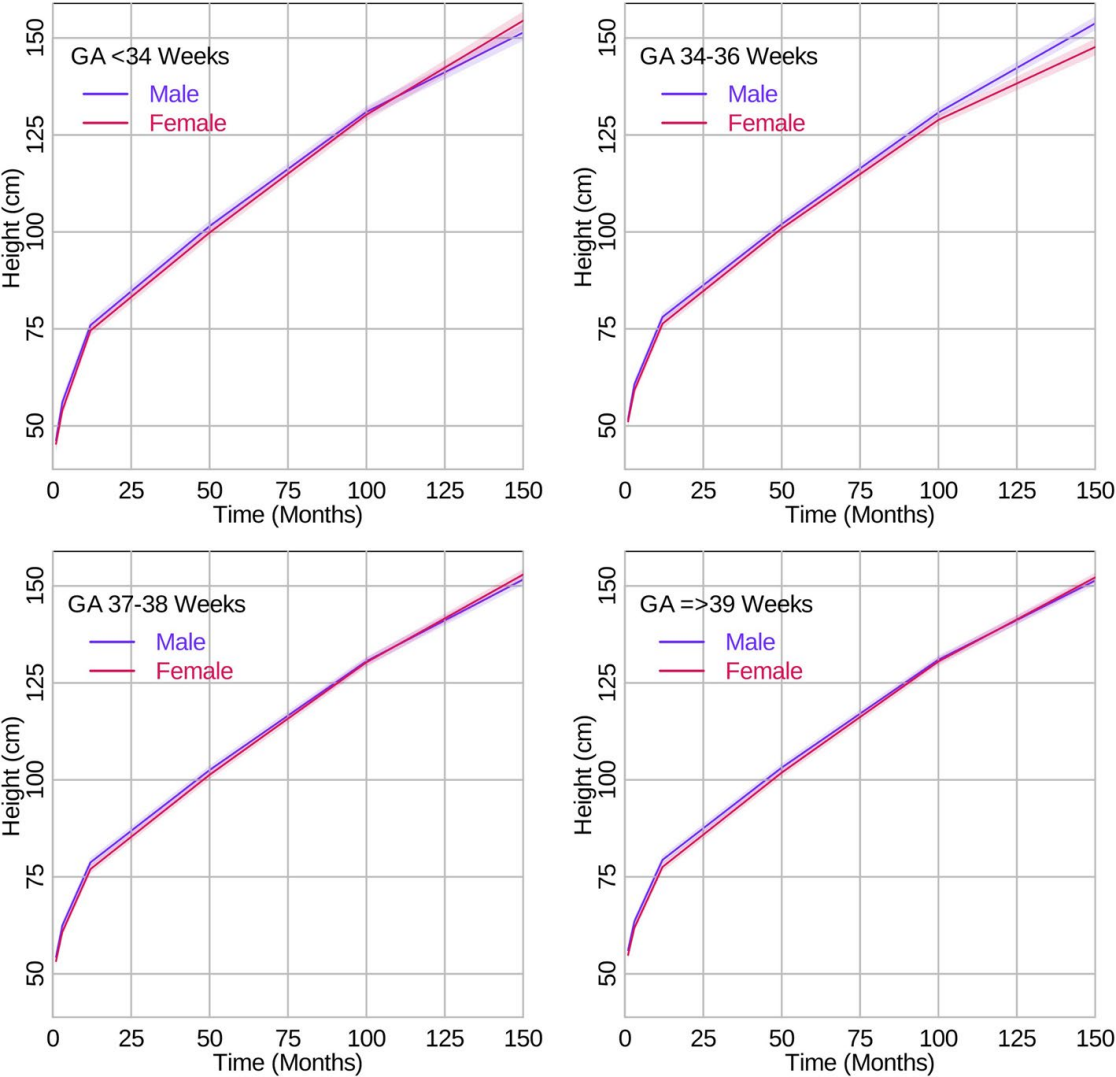
Growth curves for height trajectories in cm per month from 0 to 150 months by sex and gestational age categories. The lines represent the growth curve, and the shaded areas represent the 95% confidence intervals

Regarding the rate of height growth, for late preterm compared to full term, there did not appear to be differences by sex until 84–168 months where late preterm boys (MD 0.05 cm/month, 95% CI 0.03, 0.08) had a faster rate of growth in height than girls (MD -0.06 cm/month, 95% CI -0.09, -0.03). For very/moderate preterm compared to full term, there was some evidence that boys (MD 0.05 cm/month, 95% CI 0.03, 0.06) had a faster rate of growth in height than girls (MD 0.02 cm/month, 95% CI 0.01, 0.04) from 12–84 months. From 84–168 months, there was some evidence that girls (MD 0.05 cm/month, 95% CI 0.02, 0.09) had a faster rate of growth in height than boys (MD 0.00 cm/month, 95% CI -0.03, 0.03). For early term compared to full term, there were no notable differences between girls and boys when comparing weight or height growth rates. The results for height growth are presented in Fig. 4 and Table 3.

## Discussion

Using chronological age, our study results suggest that the mean growth trajectories of children born late preterm and very/moderate preterm differed from that of full term children with the highest degree of differences occurring between very/moderate preterm and full term children. Generally, the more preterm, the lower the mean value of weight and height until early adolescent years. Children born late preterm compared to full term had a slightly slower rate of weight gain after 84 months. The association was stronger in girls than boys. In children born late preterm, differences in height trajectories were observed from 0–12 months of age and did not appear to differ by sex. Children born very/moderate preterm had a slight increased rate of weight gain from 12–84 months but slower rate after 84 months with the slowdown more evident in boys. Further, children born very/moderate preterm had an increased rate of height gain across the age range studied, which was more evident in boys between 12–84 months and more evident in girls between 84–168 months.

Overall the rates of growth observed in our study for full term children were quite similar to previous data from four international birth cohorts which were mainly general population studies [12]. Howe et al. reported that cohorts displayed rapid weight and height gain in early infancy followed by slower growth in later infancy and early childhood [12]. For the Avon Longitudinal Study of Parents and Children (ALSPAC) cohort, where data was available for older ages, rates of weight gain began to increase again from 7 years of age which coincided with our data [12].

Few studies have investigated the association between late preterm birth and weight and height growth trajectories. A Dutch cohort study of 2324 children followed from birth to 4 years of age, reported that increases in weight and height were similar for children of different gestational ages, indicating no catch-up growth [28]. Although growth patterns were the same for boys and girls, boys demonstrated greater variability in growth by gestational age, especially at lower gestational ages (≤ 30 weeks) suggesting that preterm boys may be more susceptible than girls to complications of preterm birth that influence growth [28]. A Brazilian population-based cohort study of 3,285 children followed from birth to 24 months, reported that children born late preterm had similar weight gain to full term children whereas children born late preterm grew on average 1.26 cm more than full term [29]. The present study found that children born late preterm compared to full term had higher height growth of 0.70 cm per month but only from 0–3 months of age. Several studies have reported that accelerated postnatal growth in weight could increase the long-term risk of obesity and cardiovascular disease [13–15]. We found some evidence of faster postnatal growth in weight during early childhood (3–12 months) for late preterm compared to term.

Key strengths of this study included the ability to link clinical data from a large cohort of children to health administrative data from a publicly funded healthcare system to obtain valid prospectively measured data on gestational age at birth and maternal factors. Weight and height were measured objectively from birth to 14 years of age by staff trained using standardized instruments. We were able to adjust for confounding variables that are not routinely available in administrative data through linkage with cohort study questionnaire data and physical measures. This study also had several limitations including the possibility of residual confounding through the lack of variables such as feeding practices, environmental and lifestyle factors. We did not have a population-based representative sample because our study was nested within TARGet Kids! and reflects children recruited from selected primary care practices in one urban area in Canada. Selection bias is possible and the findings from this study may not be generalizable to other populations, especially as our sample had relatively high income and education compared to the overall population. Further, approximately 25% of eligible children were not included in the final sample due to missing data or because they were part of multiple births, limiting the representativeness of our study. As children born less than 32 weeks of age were generally excluded from TARGet Kids! at enrollment, this may have disproportionately excluded preterm children with abnormal growth trajectories. Only a small number of 58 children born < 32 weeks was included in our study. Additionally, we investigated growth until 14 years of age; however, we had less follow-up data at older ages (mean follow up time: 5.9 years), which may have reduced the precision of our estimates for older children. Since we were interested in understanding if categories of preterm children grew differently than full term, we did not correct for gestational age which may mask the differences in growth and is consistent with the methods in previous cohort studies. While it may be important to correct for gestational age when evaluating clinical outcomes for children born preterm, for this research study, we were specifically interested in understanding the differences in growth measures between preterm and full term and did not adjust away the differences by correcting for gestational age [27, 30].

## Conclusions

We found that late preterm birth was associated with a slower rate of weight gain after 84 months and faster rate of height gain from 0–12 months compared to full term birth. Follow-up is needed to determine if differences influence long-term disease risk. Advances in neonatal and pediatric care in the past few decades mean that a substantial proportion of children born late preterm are now reaching young to mid-adulthood. There is an urgent need to understand how late preterm birth contributes to increased cardiometabolic disease incidence. More data from larger studies in diverse populations may help to understand the association between late preterm birth and growth trajectories and provide clinical guidance for developing appropriate weight and height gain standards for this large subset of preterm infants.

### Supplementary Information

**Additional file 1: Supplemental Table 1.** Data sources used to define study participant characteristics and variable operationalization.** Supplemental Table 2.** Model fit comparison of predicted values at specific time points for the outcome of weight trajectory between the original model (3 knot) and model with the best fit (4 knot).** Supplemental Table 3.** Model fit comparison of predicted values at specific time points for the outcome of height trajectory between the original model (3 knot) and model with the best fit (4 knot).** Supplemental Table 4.** Predicted mean weight (kg) and 95% CI at each age (knot point) by gestational age category.** Supplemental Table 5.** Predicted mean height (cm) and 95% CI at each age (knot point) by gestational age category.** Supplemental Table 6.** Mean differences in monthly growth rates for zweight, zheight, and zBMI by gestational age categories during each growth period for study participants (*N*=4423).** Supplemental Figure 1.** Flowchart of study participants with weight/height data.** Supplemental Figure 2.** Growth curves for World Health Organization (WHO) standardized weight trajectories from 0 to 150 months by gestational age categories. The lines represent the growth curve, and the shaded areas represent the 95% confidence intervals.** Supplemental Figure 3.** Growth curves for World Health Organization (WHO) standardized height trajectories from 0 to 150 months by gestational age categories. The lines represent the growth curve, and the shaded areas represent the 95% confidence intervals.** Supplemental Figure 4.** Growth curves for World Health Organization (WHO) standardized BMI trajectories from 0 to 150 months by gestational age categories. The lines represent the growth curve, and the shaded areas represent the 95% confidence intervals.

## Data Availability

The dataset from this study is held securely in coded form at ICES. While data sharing agreements prohibit ICES from making the dataset publicly available, access may be granted to those who meet pre-specified criteria for confidential access, available at www.ices.on.ca/DAS. The full dataset creation plan and underlying analytic code are available from the authors upon request, understanding that the computer programs may rely upon coding templates or macros that are unique to ICES and are therefore either inaccessible or may require modification. Please contact Laura N. Anderson (ln.anderson@mcmaster.ca) for further information.

## Abbreviations

BORN: Better Outcomes Registry and Network
CI: Confidence Interval
CM: Centimeters
KG: Kilograms
LOESS: Locally estimated scatterplot smoothing
MD: Mean Difference
MOMBABY: Mother-Baby
TARGet Kids: The Applied Research Group for Kids
