# Supplementary material for: Late preterm birth and growth trajectories during childhood: a linked retrospective cohort study
Source: BMC Pediatr. 2023 Sep 8;23:450. doi: 10.1186/s12887-023-04257-x (PMC10485950; doi:10.1186/s12887-023-04257-x)
Supplement: Supplementary file 1 — Additional file 1: Supplemental Table 1. Data sources used to define study participant characteristics and variable operationalization. Supplemental Table 2. Model fit comparison of predicted values at specific time points for the outcome of weight trajectory between the original model (3 knot) and model with the best fit (4 knot). Supplemental Table 3. Model fit comparison of predicted values at specific time points for the outcome of height trajectory between the original model (3 knot) and model with the best fit (4 knot). Supplemental Table 4. Predicted mean weight (kg) and 95% CI at each age (knot point) by gestational age category. Supplemental Table 5. Predicted mean height (cm) and 95% CI at each age (knot point) by gestational age category. Supplemental Table 6. Mean differences in monthly growth rates for zweight, zheight, and zBMI by gestational age categories during each growth period for study participants (N=4423). Supplemental Figure 1. Flowchart of study participants with weight/height data. Supplemental Figure 2. Growth curves for World Health Organization (WHO) standardized weight trajectories from 0 to 150 months by gestational age categories. The lines represent the growth curve, and the shaded areas represent the 95% confidence intervals. Supplemental Figure 3. Growth curves for World Health Organization (WHO) standardized height trajectories from 0 to 150 months by gestational age categories. The lines represent the growth curve, and the shaded areas represent the 95% confidence intervals. Supplemental Figure 4. Growth curves for World Health Organization (WHO) standardized BMI trajectories from 0 to 150 months by gestational age categories. The lines represent the growth curve, and the shaded areas represent the 95% confidence intervals. [file 12887_2023_4257_MOESM1_ESM.docx]

Supplementary

Supplemental Table 1: Data sources used to define study participant characteristics and variable operationalization

| Characteristic | Data Source | Operationalization |
| --- | --- | --- |
| **Maternal** |  |  |
| Age at delivery | MOMBABY | Continuous in years |
| Ethnicity | TARGet Kids! | African/Arab/Latin American/Mixed Ethnicity; East/Southeast/South Asian; and European |
| **Child** |  |  |
| Gestational age | MOMBABY + BORN | Categorical (<34, 34-36, 37-38, and ≥39 weeks) |
| Age at outcome | TARGet Kids! | Continuous in years |
| Sex | TARGet Kids! | Female; male |
| Weight | TARGet Kids! | Continuous in kg |
| Height | TARGet Kids! | Continuous in cm |
| **Sociodemographic** |  |  |
| Family income | TARGet Kids! | Less than $49,000; $50, 000 to $99, 999; $100, 000 to $149, 999; $150, 000 or more |
| BORN: The Better Outcomes Registry & Network, MOMBABY: Mother-Baby Linked Dataset | | |

Supplemental Table 2: Model fit comparison of predicted values at specific time points for the outcome of weight trajectory between the original model (3 knot) and model with the best fit (4 knot).

| **Parameter** | **Estimate (SE) of 3 Knot model (knots= 3,12,84)** | **Estimate (SE) of 4 Knot model (knots= 2,8,12,84)** | **Difference between Estimates of Model 1 and Model 2 (SE)** | **Difference between SE of Model 1 and Model 2** |
| --- | --- | --- | --- | --- |
| Very/moderate preterm |  |  |  |  |
| 1 month | 2.319 (0.424) | 2.23 (0.61) | 0.089 (0.743) | -.18601 |
| 3 months | 4.327 (0.838) | 3.787 (0.907) | 0.539 (1.235) | -.06947 |
| 12 months | 8.186 (0.378) | 7.883 (0.43) | 0.303 (0.573) | -.05151 |
| 50 months | 15.828 (0.338) | 15.859 (0.343) | -0.031 (0.482) | -.00564 |
| 100 months | 27.188 (0.379) | 27.126 (0.383) | 0.062 (0.539) | -.00399 |
| 150 months | 41.321 (0.594) | 41.333 (0.595) | -0.012 (0.841) | -.00044 |
| Late preterm |  |  |  |  |
| 1 month | 3.122 (0.338) | 3.126 (0.412) | -0.004 (0.532) | -.07408 |
| 3 months | 4.863 (0.564) | 4.699 (0.559) | 0.164 (0.794) | .00535 |
| 12 months | 9.384 (0.317) | 8.987 (0.348) | 0.397 (0.471) | -.03022 |
| 50 months | 16.386 (0.296) | 16.411 (0.298) | -0.025 (0.42) | -.00213 |
| 100 months | 26.867 (0.324) | 26.815 (0.326) | 0.053 (0.46) | -.00198 |
| 150 months | 40.042 (0.534) | 40.066 (0.536) | -0.024 (0.756) | -.00149 |
| Early term |  |  |  |  |
| 1 month | 3.785 (0.289) | 3.845 (0.325) | -0.06 (0.435) | -.03624 |
| 3 months | 5.627 (0.406) | 5.39 (0.404) | 0.238 (0.573) | .00185 |
| 12 months | 9.533 (0.276) | 9.084 (0.287) | 0.449 (0.399) | -.01104 |
| 50 months | 16.636 (0.27) | 16.691 (0.27) | -0.055 (0.382) | -.00046 |
| 100 months | 27.922 (0.276) | 27.844 (0.277) | 0.078 (0.391) | -.00066 |
| 150 months | 43.332 (0.335) | 43.39 (0.335) | -0.057 (0.473) | -.00031 |
| Full term |  |  |  |  |
| 1 month | 4.115 (0.266) | 4.195 (0.285) | -0.081 (0.39) | -.01829 |
| 3 months | 5.961 (0.313) | 5.717 (0.323) | 0.244 (0.45) | -.00979 |
| 12 months | 9.737 (0.263) | 9.218 (0.267) | 0.518 (0.374) | -.00408 |
| 50 months | 16.76 (0.26) | 16.829 (0.26) | -0.069 (0.368) | -.00028 |
| 100 months | 27.95 (0.263) | 27.864 (0.263) | 0.086 (0.371) | -.00021 |
| 150 months | 43.277 (0.29) | 43.364 (0.291) | -0.087 (0.411) | -.00028 |

Supplemental Table 3: Model fit comparison of predicted values at specific time points for the outcome of height trajectory between the original model (3 knot) and model with the best fit (4 knot).

| **Parameter** | **Estimate (SE) of 3 Knot model (knots= 3,12,84)** | **Estimate (SE) of 4 Knot model (knots= 4,12,24,84)** | **Difference between Estimates of Model 1 and Model 2 (SE)** | **Difference between SE of Model 1 and Model 2** |
| --- | --- | --- | --- | --- |
| Very/moderate preterm |  |  |  |  |
| 1 month | 45.811 (0.783) | 45.906 (0.704) | -0.095 (1.053) | .079038 |
| 3 months | 54.96 (0.58) | 54.005 (0.547) | 0.955 (0.797) | .032581 |
| 12 months | 75.232 (0.532) | 73.585 (0.565) | 1.647 (0.776) | -.033058 |
| 50 months | 100.624 (0.518) | 101.045 (0.52) | -0.42 (0.734) | -.001813 |
| 100 months | 130.39 (0.555) | 129.803 (0.557) | 0.588 (0.787) | -.001580 |
| 150 months | 152.412 (0.794) | 152.816 (0.777) | -0.403 (1.111) | .016487 |
| Late preterm |  |  |  |  |
| 1 month | 51.307 (0.493) | 51.257 (0.489) | 0.05 (0.694) | .003873 |
| 3 months | 59.87 (0.49) | 58.87 (0.471) | 1 (0.68) | .019309 |
| 12 months | 77.2 (0.466) | 75.141 (0.483) | 2.059 (0.671) | -.017357 |
| 50 months | 101.387 (0.459) | 101.923 (0.459) | -0.537 (0.649) | -.000777 |
| 100 months | 129.908 (0.481) | 129.167 (0.482) | 0.741 (0.681) | -.000413 |
| 150 months | 151.408 (0.696) | 152.145 (0.683) | -0.737 (0.975) | .013751 |
| Early term |  |  |  |  |
| 1 month | 53.748 (0.427) | 53.646 (0.426) | 0.102 (0.604) | .000867 |
| 3 months | 61.553 (0.428) | 60.638 (0.423) | 0.915 (0.602) | .005434 |
| 12 months | 77.843 (0.422) | 75.931 (0.427) | 1.912 (0.6) | -.004707 |
| 50 months | 101.88 (0.42) | 102.399 (0.42) | -0.519 (0.594) | -.000084 |
| 100 months | 130.374 (0.425) | 129.741 (0.425) | 0.634 (0.601) | .000073 |
| 150 months | 152.214 (0.484) | 152.938 (0.48) | -0.725 (0.682) | .004144 |
| Full term |  |  |  |  |
| 1 month | 55.439 (0.409) | 55.36 (0.408) | 0.079 (0.577) | .000552 |
| 3 months | 62.609 (0.409) | 61.826 (0.406) | 0.784 (0.576) | .002446 |
| 12 months | 78.43 (0.406) | 76.436 (0.408) | 1.994 (0.575) | -.001884 |
| 50 months | 102.456 (0.405) | 102.984 (0.405) | -0.529 (0.572) | .000081 |
| 100 months | 130.671 (0.407) | 129.995 (0.407) | 0.675 (0.575) | .000182 |
| 150 months | 151.666 (0.435) | 152.446 (0.433) | -0.78 (0.614) | .002082 |

Supplemental Table 4: Predicted mean weight (kg) and 95% CI at each age (knot point) by gestational age category

| Age | Very/Moderate preterm | Late preterm | Early term | Full term |
| --- | --- | --- | --- | --- |
| 1 mo | 2.3 (1.5, 3.1) | 3.1 (2.5, 3.8) | 3.8 (3.2, 4.4) | 4.1 (3.6, 4.6) |
| 3 mo | 4.3 (2.7, 6.0) | 4.9 (3.8, 6.0) | 5.6 (4.8, 6.4) | 6.0 (5.3, 6.6) |
| 12 mo | 8.2 (7.4, 8.9) | 9.4 (8.8, 10.0) | 9.5 (9.0, 10.1) | 9.7 (9.2, 10.3) |
| 50 mo | 15.8 (15.2, 16.5) | 16.4 (15.8, 17.0) | 16.6 (16.1, 17.2) | 16.8 (16.3, 17.3) |
| 100 mo | 27.2 (26.4, 27.9) | 26.9 (26.2, 27.5) | 27.9 (27.4, 28.5) | 27.9 (27.4, 28.5) |
| 150 mo | 41.3 (40.2, 42.5) | 40.0 (39.0, 41.1) | 43.3 (42.7, 44.0) | 43.3 (42.7, 43.8) |

Supplemental Table 5: Predicted mean height (cm) and 95% CI at each age (knot point) by gestational age category

| Age | Very/Moderate preterm | Late preterm | Early term | Full term |
| --- | --- | --- | --- | --- |
| 1 mo | 45.8 (44.3, 47.3) | 51.3 (50.3, 52.3) | 53.7 (52.9, 54.6) | 55.4 (54.6, 56.2) |
| 3 mo | 55.0 (53.8, 56.1) | 59.9 (58.9, 60.8) | 61.6 (60.7, 62.4) | 62.6 (61.8, 63.4) |
| 12 mo | 75.2 (74.2, 76.3) | 77.2 (76.3, 78.1) | 77.8 (77.0, 78.7) | 78.4 (77.6, 79.2) |
| 50 mo | 100.6 (99.6, 101.6) | 101.4 (100.5, 102.3) | 101.9 (101.1, 102.7) | 102.5 (101.7, 103.2) |
| 100 mo | 130.4 (129.3, 131.5) | 129.9 (129.0, 130.9) | 130.4 (129.5, 131.2) | 130.7 (129.9, 131.5) |
| 150 mo | 152.4 (150.9, 154.0) | 151.4 (150.0, 152.8) | 152.2 (151.3, 153.2) | 151.7 (150.8, 152.5) |

Supplemental Table 6: Mean differences in monthly growth rates for zweight, zheight, and zBMI by gestational age categories during each growth period for study participants (N=4423)

| Growth period | Growth rate (95% CI) very/moderate preterm | Mean difference (95% CI) between very/moderate preterm vs. full term | Growth rate (95% CI) late preterm | Mean difference (95% CI) between late preterm vs. full term | Growth rate (95% CI) early term | Mean difference (95% CI) between early term vs. full term | Growth rate (95% CI) full term |
| --- | --- | --- | --- | --- | --- | --- | --- |
| zWeight | | | | | | | |
| 0-3 months | 0.22 (0.15, 0.29) | 0.22 (0.15, 0.29) | 0.00 (0.00, 0.00) | 0.00 (-0.01, 0.00) | 0.00 (-0.01, 0.01) | 0.00 (-0.01, 0.01) | 0.00 (0.00, 0.01) |
| 3-12 months | 0.27 (0.25, 0.28) | 0.23 (0.21, 0.24) | 0.17 (0.16, 0.18) | 0.13 (0.12, 0.14) | 0.08 (0.08, 0.08) | 0.04 (0.03, 0.04) | 0.04 (0.04, 0.05) |
| 12-84 months | 0.01 (0.00, 0.01) | 0.01 (0.00, 0.01) | 0.00 (0.00, 0.00) | 0.00 (0.00, 0.00) | 0.00 (0.00, 0.00) | 0.00 (0.00, 0.00) | 0.00 (0.00, 0.00) |
| 84-168 months | 0.00 (-0.01, 0.01) | -0.01 (-0.01, 0.00) | 0.01 (0.00, 0.01) | 0.00 (0.00, 0.01) | 0.00 (0.00, 0.01) | 0.00 (0.00, 0.00) | 0.00 (0.00, 0.01) |
| zHeight | | | | | | | |
| 0-3 months | 0.16 (0.07, 0.25) | 0.14 (0.04, 0.23) | 0.00 (0.00, 0.00) | -0.02 (-0.03, -0.02) | 0.02 (0.01, 0.03) | 0.00 (-0.01, 0.01) | 0.02 (0.02, 0.03) |
| 3-12 months | 0.22 (0.21, 0.24) | 0.26 (0.24, 0.28) | 0.11 (0.10, 0.12) | 0.14 (0.13, 0.16) | 0.02 (0.01, 0.02) | 0.05 (0.05, 0.06) | -0.04 (-0.04, -0.03) |
| 12-84 months | 0.01 (0.01, 0.01) | 0.01 (0.01, 0.02) | 0.00 (0.00, 0.00) | 0.00 (0.00, 0.01) | 0.00 (-0.01, 0.00) | 0.00 (0.00, 0.00) | -0.01 (-0.01, -0.01) |
| 84-168 months | 0.00 (0.00, 0.01) | 0.00 (-0.01, 0.00) | 0.01 (0.00, 0.01) | 0.00 (0.00, 0.01) | 0.01 (0.01, 0.01) | 0.00 (0.00, 0.00) | 0.01 (0.01, 0.01) |
| zBMI | | | | | | | |
| 0-3 months | 0.14 (0.04, 0.23) | 0.16 (0.06, 0.25) | 0.00 (0.00, 0.00) | 0.02 (0.01, 0.02) | -0.01 (-0.02, -0.01) | 0.01 (0.00, 0.01) | -0.02 (-0.02, -0.01) |
| 3-12 months | 0.18 (0.16, 0.20) | 0.09 (0.07, 0.11) | 0.15 (0.14, 0.16) | 0.06 (0.05, 0.07) | 0.10 (0.09, 0.10) | 0.01 (0.00, 0.02) | 0.09 (0.08, 0.09) |
| 12-84 months | 0.00 (0.00, 0.00) | 0.00 (0.00, 0.00) | 0.00 (0.00, 0.00) | 0.00 (0.00, 0.00) | 0.00 (0.00, 0.00) | 0.00 (0.00, 0.00) | 0.00 (0.00, 0.00) |
| 84-168 months | 0.00 (-0.01, 0.00) | 0.00 (-0.01, 0.00) | 0.00 (-0.01, 0.00) | 0.00 (-0.01, 0.00) | 0.00 (-0.01, 0.00) | 0.00 (0.00, 0.00) | 0.00 (0.00, 0.00) |

10 897 children in TARGet Kids! data

5907 potentially eligible children

4990 without a valid ICES-specific key number or with birthdate < April 1, 2006, or > September 30, 2014

108 who were not Ontario residents or were not eligible for Ontario’s health care plan (OHIP) on their birthdate

5799 potentially eligible children

770 with no weight measurement, only one weight measurement, impractical weight, or impractical zBMI values

4423 final linked TARGet Kids! – ICES – BORN cohort

311 missing gestational age data

4718 potentially eligible children

295 who were twins, triplets, or part of other multiple birth

5029 potentially eligible children

Supplemental Figure 1: Flowchart of study participants with weight/height data


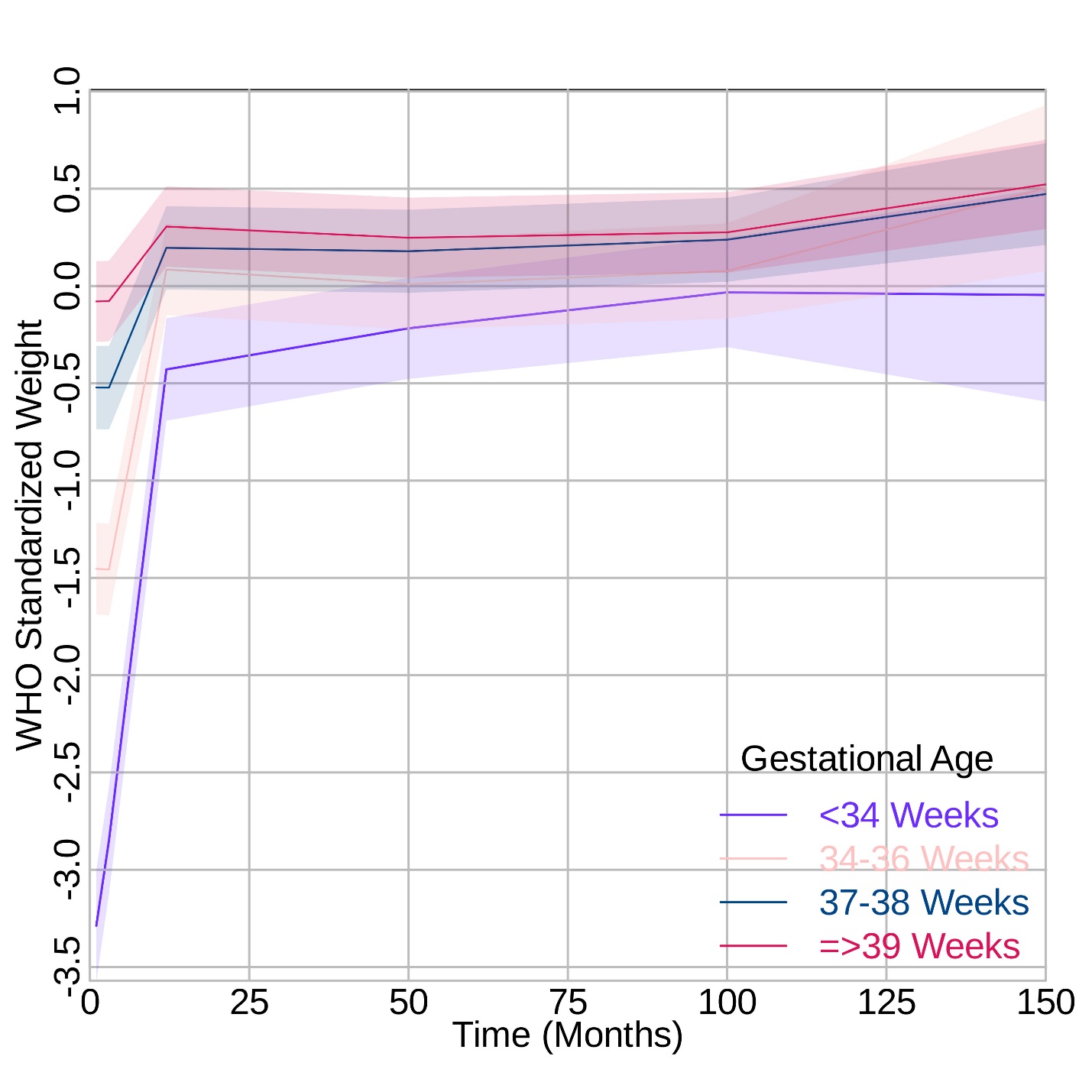


Supplemental Figure 2: Growth curves for World Health Organization (WHO) standardized weight trajectories from 0 to 150 months by gestational age categories. The lines represent the growth curve, and the shaded areas represent the 95% confidence intervals.


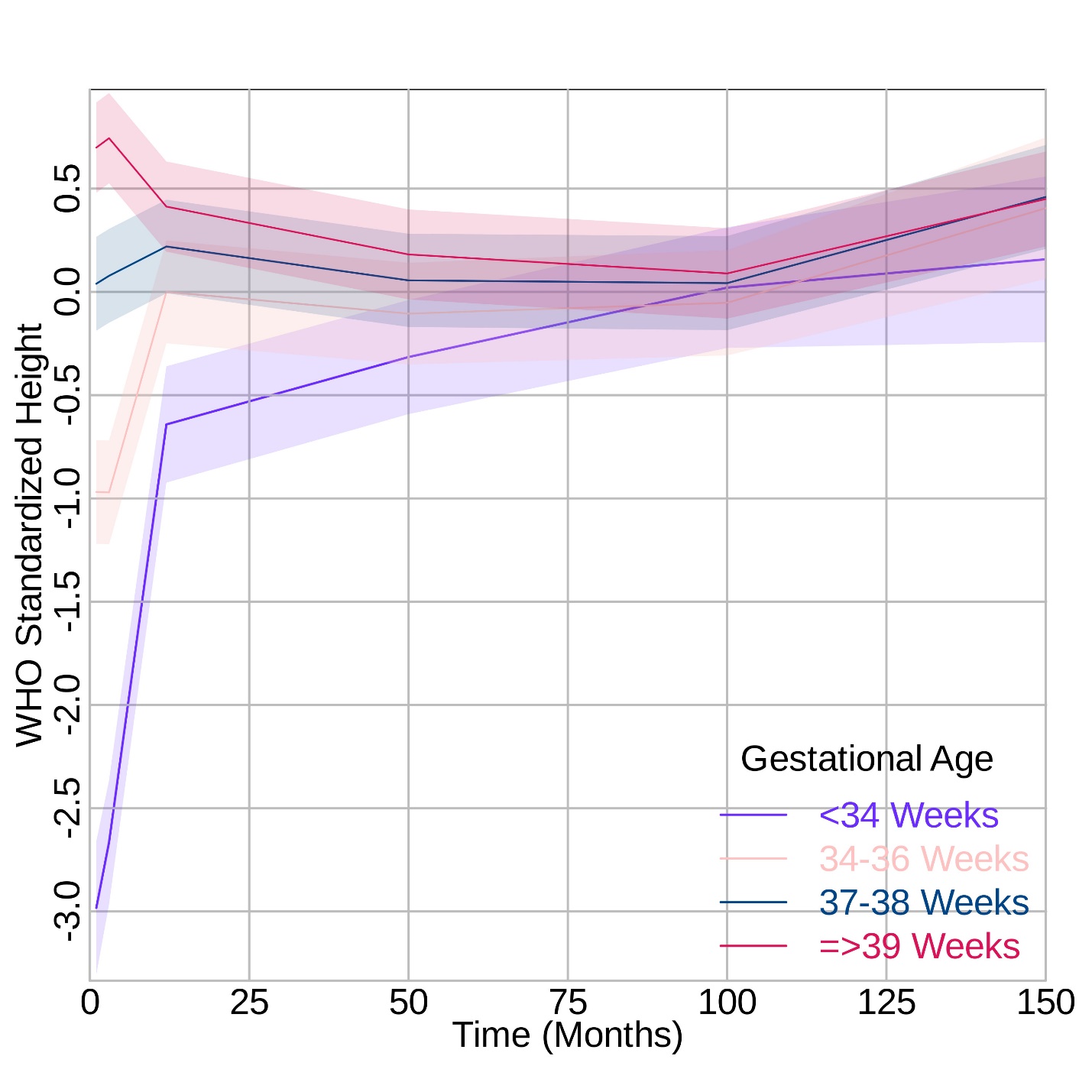


Supplemental Figure 3: Growth curves for World Health Organization (WHO) standardized height trajectories from 0 to 150 months by gestational age categories. The lines represent the growth curve, and the shaded areas represent the 95% confidence intervals.


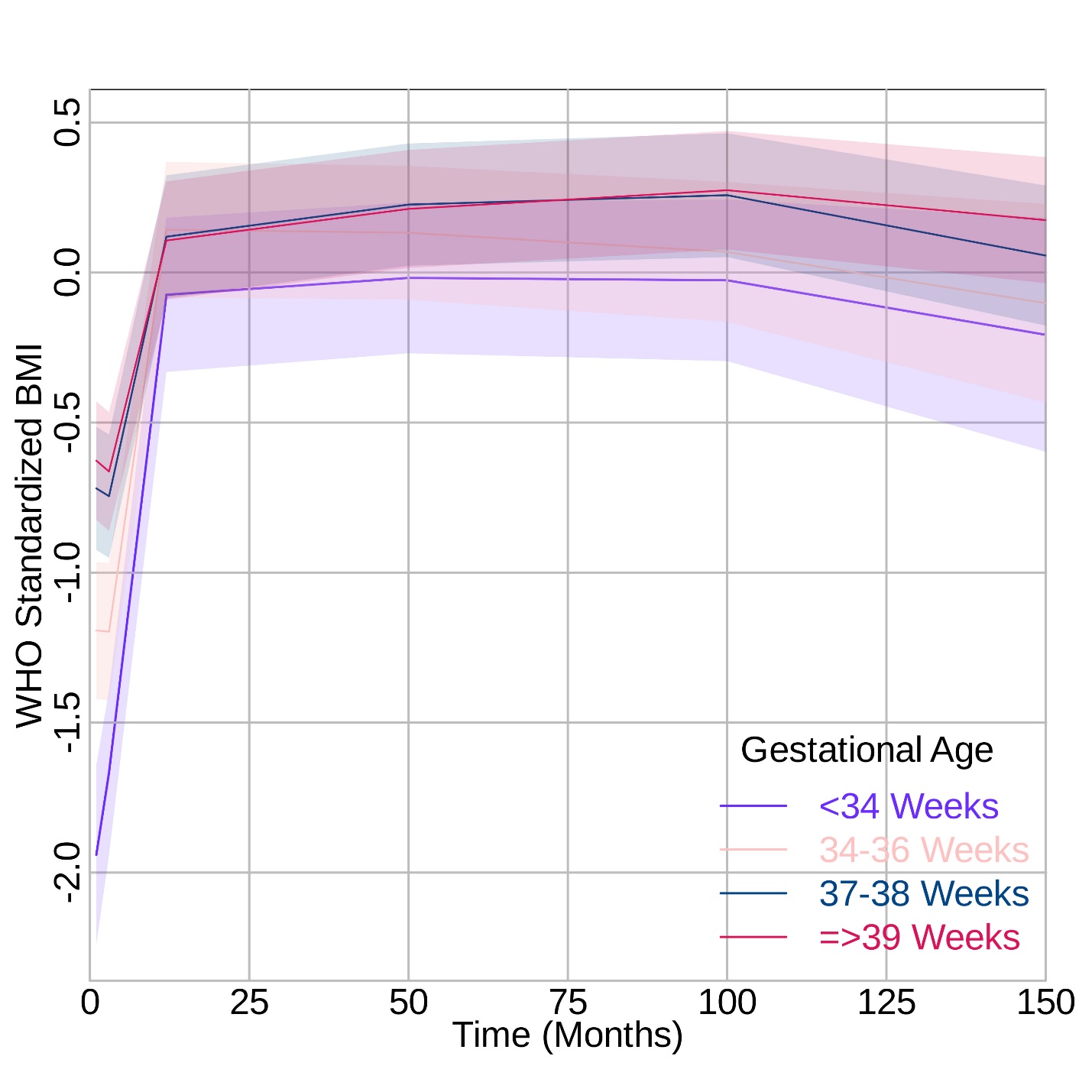


Supplemental Figure 4: Growth curves for World Health Organization (WHO) standardized BMI trajectories from 0 to 150 months by gestational age categories. The lines represent the growth curve, and the shaded areas represent the 95% confidence intervals.
